# ACL reconstruction provides superior stability than ACL repair in patients with Schenck III and IV knee joint dislocations: first results of a 12 month follow-up study

**DOI:** 10.1007/s00402-023-04884-0

**Published:** 2023-04-16

**Authors:** H. Fahlbusch, P. Behrendt, R. Akoto, K. H. Frosch, M. Krause

**Affiliations:** 1 Department of Trauma and Orthopaedic Surgery, University Medical Center Hamburg-Eppendorf, Hamburg, Germany; 2 Department of Trauma Surgery, Orthopaedics and Sports Orthopaedics, Asklepios St. Georg, Hamburg, Germany; 3 Department of Anatomy, Christian-Albrechts-University, Kiel, Germany; 4 Department of Trauma Surgery, Orthopaedics and Sports Traumatology, BG Hospital Hamburg, Hamburg, Germany

**Keywords:** Knee dislocation, Internal bracing, Knee dislocation, Multiligament injury, Internal bracing, ACL reconstruction

## Abstract

**Purpose:**

Acute knee dislocation is a rare but devastating multi-ligamentous knee injury with only limited evidence-based surgical technique recommendations. The aim of this study was a comparison of two different anterior cruciate ligament (ACL) restoration techniques as part of an early total surgical care concept: (1) repair of ACL with additional internal bracing (ACLIB) compared to; (2) ACL reconstruction with autograft (ACLR).

**Methods:**

Retrospective, clinical-study of patients with an acute type III or IV knee dislocation (according to Schenck classification), in which the ACL was treated with ACLIB or ACLR within 12 days. The PCL was sutured and internally braced in all cases. Medial and lateral complex injuries were repaired and additionally laterally augmented by an Arciero reconstruction. After a minimum 12 months follow-up different patient-reported outcome measurements (IKDC, Lysholm, VAS, Tegner Score) and instrumental stability assessment by Rolimeter -test and stress radiographs (Telos^™^) were analyzed. Groups were compared by *t* test with *p* < 0.05 considered significant.

**Results:**

In total, 20 patients (5 IIIM, 5 IIIL and 10 IV) were included in this study with an average follow-up of 13.7 ± 2.6 months. There were significant differences in instrumental stability testing (side-to-side difference (SSD) of anterior tibial translation: ACLIB 2.7 ± 1.5 mm vs. ACLR 1.3 ± 1.3; *p* = 0.0339) and stress radiography (SSD ACL: ACLIB 3.4 ± 2.2 mm vs. ACLR 0.4 ± 2.7; *p* = 0.0249) between groups. ACLIB group showed greater ROM in terms of flexion (SSD Flexion: ACLIB 7.8 ± 9.9° vs. ACLR 16 ± 7.0°; *p* = 0.0466; Total Flexion overall 125.5 ± 11.8°). No clinically relevant differences in patient-reported outcome scores (Lysholm Score: ACLIB 82 ± 16.4 vs. ACLR 85 ± 10.4; IKDC subjective score: ACLIB 70.4 ± 17 vs. ACLR 76.6 ± 8.3) were determined.

**Conclusion:**

ACLR provides superior translational stability than ACLIB in terms of instrumental testing and stress radiography. Both techniques were equivalent with respect to PROMS and led to good and excellent clinical results.

**Level of evidence:**

Retrospective cohort study, III.

## Introduction

Acute knee dislocations (KD) are rare but devastating knee injuries. Nonsurgical therapy yields unsatisfactory results and significantly impairs the quality of life due to pain and instability [25, 30, 32]. Surgical treatment on the other hand is demanding and various surgical techniques have been proposed [12, 25]. These range from early to late surgery and repair versus reconstruction. Surgery can be realized in a single-stage or two-stage procedure [5]. Due to the inhomogeneity of injury patterns, small case numbers and various associated injuries, evidence-based treatment recommendations are missing and treatment options are controversially discussed.

Frosch et al. reported in a meta-analysis that acute suture repair yielded good clinical results, which are comparable to those of ligament reconstructions [12]. In continuation, the concept of additional internal bracing was proposed and realized in an early total repair technique of acute KD as described by Heitmann et al. [16]. Applying this technique, a multicentre study demonstrated promising results of this “ligament bracing” surgical technique [19]. Nevertheless, stability assessment following ligament bracing still does not match values of single ACL reconstruction. Clinical results showed residual laxity and failure rates of up to 17% [19, 33], mainly due to ACL repair. In this regard, a failure rate of 16% has been reported for primary single ACL repair and internal bracing at a two year follow-up [24], which has recently been reported to increase up to 28% at a 5 year follow-up [13]. Therefore, it must be questioned, whether ACL ligament repair is equally successful compared to primary ACL reconstruction in acute KD. Additional harvesting and anchoring of autologous tendon grafts certainly carry the potential for complications and thus could also have an impact on clinical outcome. Therefore, the question if ligament repair or reconstruction provides greater stability and better outcomes remains controversial [6, 12, 28, 33]. The aim of this study was compare the concept of ACL repair with an additional internal bracing (ACLIB) in cases of acute KD against ACL autograft reconstruction (ACLR). We hypothesized superiority of ACLR over ALCIB in restoring anterior tibial translation and a better overall clinical outcome despite possible donor site morbidity.

## Materials and methods

Patient population: the study design was approved by the local ethics committee and an informed consent was obtained by each patient (2020-10227-BO-ff). All patients were informed about the treatment options and agreed preoperatively to the elucidated procedure.

Between 2018 until 2021, 23 patients with acute knee were included in a retrospective cohort study. The treatment of multiligamentary knee injuries was modified over time according to our clinical experience. Between 2018 and 2020 ACL repair with additional bracing was performed. From 2020 onward, the standard therapy changed to ACL reconstruction using hamstring tendon autografts.

Knee dislocation was categorized according to the classification reported by Schenck et al. [35]. Only patients presenting with clinical and radiological evidence of an acute type III or IV KD were included in this study. Exclusion criteria were an age under 18, polytraumatized patients, popliteal artery injuries, chronic injuries (older 12 days), peroneal nerve injuries and ultra-low velocity injuries in obese patients (grade II according to WHO definition BMI > 35 km/m^2^.

**Fig. 1:**
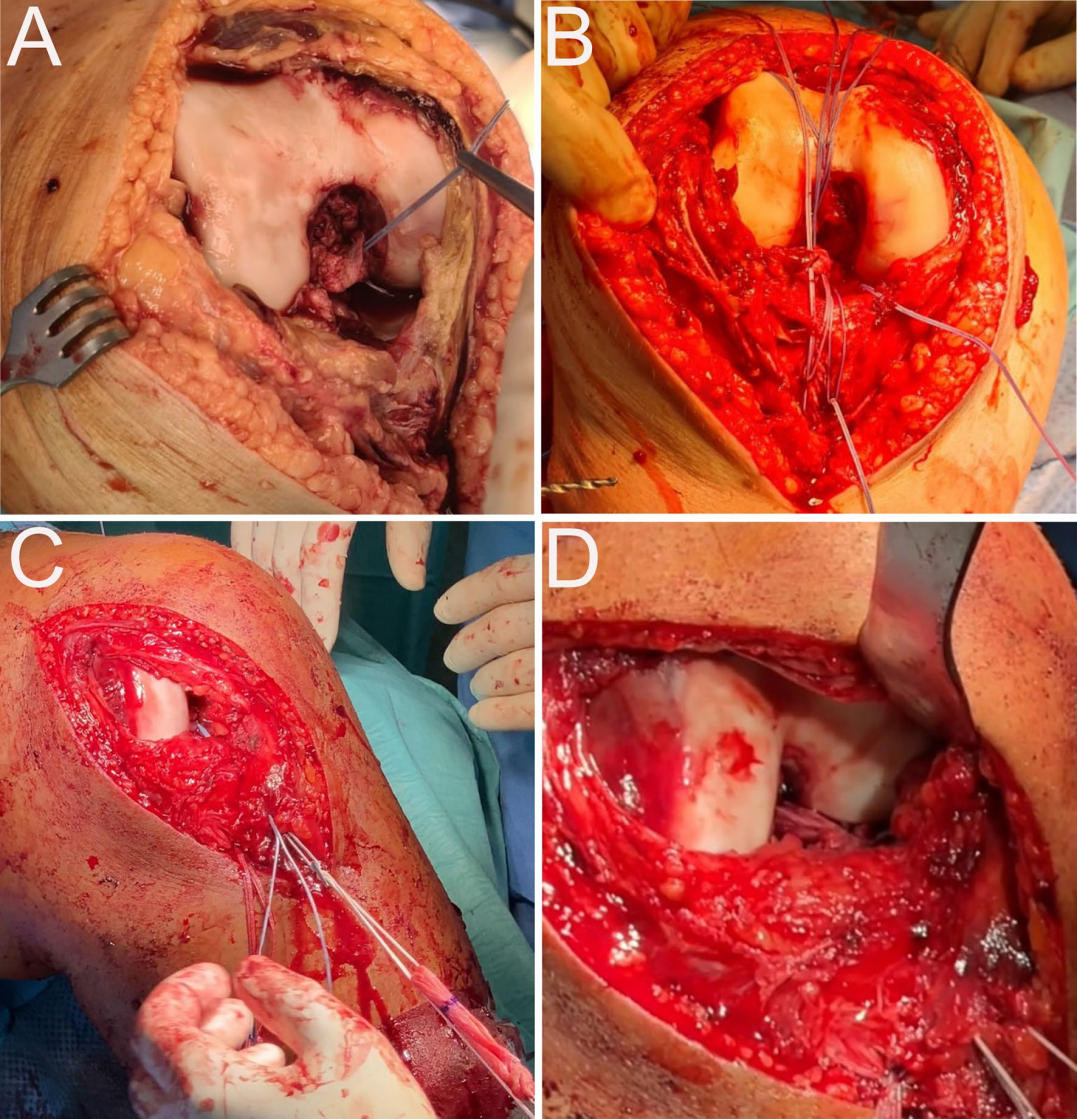
Treatment strategies for the ACL in acute KD. **A**, **B** Case of a Schenck IV patient who received an ACL suture and augmentation using an internal brace. Femoral ACL stump armed with sutures for transosseous fixation (**A**) and additionally braced with a highly durable suture (ligament bracing, **B**). **C**, **D** Case of a Schenck IV patient who received an ACLR. An autologous semitendinosus tendon graft was shuttled into the tibial drilling canal **C**. ACL autograft in situ after tibial and femoral fixation **D**

Surgical management: Surgical management was based on plain radiographs, MRI scans, physical examination of ligamentous instability and intraoperative findings. The surgical technique used for ACLIB was described in detail before [4, 16, 19]. First, a short arthroscopy is performed to address meniscal tears and possible associated chondral lesions. Meniscal repair was suitable in all cases. Subsequently open restoration of all torn ligaments was performed. In case of a type IIIM acute KD an anteromedial parapatellar arthrotomy and in case of IIIL or KD IV an additional lateral incision was performed to address the posterolateral corner. The ligament stumps of the cruciate ligaments were armed with type 2 FiberWire#2^®^ (Arthrex) for transosseous fixation. ACL and posterior cruciate ligament (PCL) tunnels were drilled in standard positions using the assistance of arthroscopic ACL and PCL drill guides. After preparation of any drill tunnels used for peripheral reconstructions, first the PCL was reattached to its footprint by tensioning the armed sutures and additional internal brace augmentation using a FiberTape^®^ (Arthrex) suture at 70°–90° of knee flexion and fluoroscopic control. Thereafter, the ACL was reattached accordingly in 20°–30° flexion and augmented using an internal brace (Fig. 1A, B). Augmentation and pull-out sutures were extracortically knotted using metal suture buttons.

In cases with ACLR, ACL target wires for the subsequent drill channels were placed arthroscopically, since from the authors’ point of view the insertion anatomy of the ACL in the open preparation may be complicated by the lateralized patella and the subluxated tibial head. All ACLR were performed using a single-bundle hamstring and anteromedial portal drilling technique (Fig. 1C, D). For anatomical footprint ACL reconstruction, the anteromedial portion of the ACL was aimed the femoral side [29] and at the tibia 43% of the antero (0%)-posterior (100%) tibial diameter was aimed [39].

After restoration of the cruciate ligaments the peripheral ligaments were addressed according to the concept “repair what is torn”. Intraligamentous injuries were sutured whenever possible. In posteromedial injuries with avulsion of the superficial medial collateral ligament (sMCL) femoral suture anchors in combination with a capsule duplication in the technique according to Hughston et al. [20] were performed. In cases of posterolateral corner injuries with injury of the popliteus tendon, a reconstruction based on Arciero’s technique with a hamstring tendon autograft was performed.

Rehabilitation: In all cases peripheral nerve block anesthesia was applied. Standardized physical therapy started 48 h after the operation with passive motion of the joint in the prone position with limited range of motion (ex./flex. 0°/0°/90°). Patients had limited weight bearing at a maximum of 20 kg for 6 weeks. Stabilizing braces without posterior tibial support were worn for 12 weeks, limiting the range of motion for 6 weeks (ex./flex. 0/0/90°).

Clinical testing: Follow-up examination was conducted 12 month following surgery and included functional outcome scoring systems by Lysholm, Tegner, and International Knee Documentation Committee (IKDC). Time to return to sports was recorded. Subjective pain during rest and exercise was quantified by visual analogous scale (VAS). Instrumental measurement was conducted by Rolimeter-Test (Aircast) to measure the anterior translation of the tibia. To quantify the side-to-side difference, stress radiographs (Telos^®^) of both knees with 15 kp posterior and anterior forces to the tibia in 90° of flexion were performed. In addition, range of motion (ROM), Dial test and anterior tibial translation was measured clinically with the Lachman (grade 1: 3–5 mm; grade 2: 6–10 mm; and grade 3: > 10 mm) and pivot-shift test (grade 1 = glide; grade 2 = clunk; and grade 3 = gross). Varus and valgus stability was tested at 0 and 30 degrees to evaluate the collateral ligaments.

Postoperative clinical failure was defined as non-traumatic ACL re-rupture that was validated by MRI scan or arthroscopically, or a SSD of > 6 mm in instrumental anterior stress tests.

Statistical Analysis: Data are presented as means and standard deviations (SD). The calculation was based on two groups: (1) repair of ACL with additional internal bracing (ACLIB); compared to (2) ACL reconstruction with autograft (ACLR). Primary outcome was defined by anterior tibial translation testing and secondary outcomes by PROMs. Differences between the groups were calculated with the Student’s *t* test and Mann–Whitney *U* Test for non-parametric parameters. Categorical parameters were compared using Fisher’s exact text. Statistical analysis was performed using GraphPad Prism 8 (San Diego, CA, US). A *p* value < 0.05 was considered significant. A sample size calculation revealed *n* = 20 patients to detect 1.5 mm change in the instrumented Lachman test using G-Power (version 3.1.9.7., Heinrich Heine Universität, Düsseldorf) with a α-error of 5% and test power of 0.8 [19].

## Results

### Patient demographics

Demographic data of the included cases are displayed in Table 1. After an average follow-up of 13.7 ± 2.6 months twenty patients were ultimately included in the study. One patient was lost during follow-up (ACLIB) and two patients (each in both groups) suffered a traumatic ACL re-rupture and were not clinically assessed.

**Table 1:** Demographic data

Characteristics	Total (*n* = 20)	ACLIB‡ (*n* = 10)	ACLR‡ (*n* = 10)	*p* value‡
Female Sex†, *n* (in %)	6 (30)	4 (40)	2 (20)	0.6211
Age^§^	34.0 ± 14.2	36.6 ± 12.9	31.4 ± 15.8	0.1962
Left Knee†, *n* (%)	14 (70)	5 (50)	9 (90)	0.1409
BMI > 30 kg/m^2^†	4 (20)	3 (30)	1 (10)	0.5820
Follow-up, in months^§^	13.7 ± 2.6	14.7 ± 3	12.8 ± 2	0.1149
High velocity trauma†	9 (45)	5 (50)	4 (40)	0.9999
Schenck Classification†				n.s
Schenck IIIM	5 (25)	3 (30)	2 (20)	
Schenck IIIL	5 (25)	2 (20)	3 (30)	
Schenck IV	10 (50)	5 (50)	5 (50)	
Concomitant injuries†				n.s
Meniscal lesions	10 (50)	5 (50)	5 (50)	
Lig. Patellae	2 (10)	1 (10)	1 (10)	
Posterolateral Capsule or Popliteus complex	6 (30)	1 (10)	5 (50)	

Bold *p* value indicates statistical significance

*SD* standard deviation, *ACLIB* anterior cruciate ligament internal bracing, *ACLR* anterior cruciate ligament reconstruction

*n* = 20

^§^Mean ± SD

^†^*n* (in %)

^‡^Shapiro–Wilk normality test and Kolmogorov–Smirnov test were performed to determine if the data were normally distributed, To compare ACLIB and ACLR Student’s *t* test or Mann–Whitney *U* test were performed, Fisher’s exact test was used for comparison of binominal data

### Patient reported functional outcome

Functional outcome scores at the time of follow-up are given in Table 2. There was no difference between the two groups according to VAS, Tegner score, Return to sports and functional scores.

**Table 2:** Functional outcome scores at the follow-up

Parameters	Total (*n* = 20)	ACLIB‡ (*n* = 10)	ACLR‡ (*n* = 10)	*p* value‡
VAS rest^§^	0.6 ± 1.1	0.6 ± 1.3	0.6 ± 1.1	0.9999
VAS exercise	2.5 ± 2	2.7 ± 1.5	2.3 ± 2.5	0.6515
Lysholm Score^§^	83.5 ± 13.5	82 ± 16.4	85 ± 10.4	0.7591
Subjective IKDC score^§^	73.5 ± 13.4	70.4 ± 17	76.6 ± 8.3	0.3189
Tegner Score^§^				
Preoperative	6.4 ± 2	6.2 ± 1.5	6.6 ± 2.4	0.6644
Postoperative	4.8 ± 1.6	4.6 ± 1.4	4.9 ± 1.8	0.6774
Delta Δ (pre-post)	1.7 ± 1.3	1.6 ± 1.4	1.7 ± 1.3	0.8655
Return to sports^§^, in months	9.8 ± 2.8 (5–12)	10.1 ± 2.6 (6 -13)	9.5 ± 3 (5–12)	0.5849

Bold *p* value indicates statistical significance

*SD* standard deviation, *ACLIB* anterior cruciate ligament internal bracing, *ACLR* anterior cruciate ligament reconstruction, *VAS* visual analogous scale, *IKDC* International Knee Documentation Committee (IKDC)

*n* = 20

^§^Mean ± SD

^†^*n* (in %)

^‡^Shapiro–Wilk normality test and Kolmogorov–Smirnov test were performed to determine if the data were normally distributed, to compare ACLIB and ACLR Student’s *t* Test or Mann–Whitney *U* Test were performed, Fisher’s exact test was used for comparison of binominal data

### Clinical testing and instrumental stability testing

Data of clinical examination and instrumental testing at the time of follow-up are given in Table 3. Significant differences were shown between groups in anterior tibial translation (SSD ACLIB 2.7 ± 1.5 mm vs. ACLR 1.3 ± 1.3; *p* = 0.0339), stress radiography (SSD ACL: ACLIB 3.4 ± 2.2 mm vs. ACLR 0.4 ± 2.7; *p* = 0.0249) and side-to-side difference of flexion (SSD Flexion: ACLIB 7.8 ± 9.9° vs. ACLR 16 ± 7.0°; *p* = 0.0466). There were no significant difference between groups in terms of extension, Varus-/Valgus thrust, Pivot-Shift and Dial test (external and internal rotation).

**Table 3:** Clinical examination and instrumental stability assessment at the follow-up

Parameters	Total (*n* = 20)	ACLIB † (*n* = 10)	ACLR † (*n* = 10)	*p* value
Flexion^§^, in °	125.5 ± 11.8	130.5 ± 12.1	120.5 ± 9.6	0.0554
Flexion SSD^§^, in °	11.90 ± 9.4	7.8 ± 9.9	16 ± 7.0	**0.0466**
Extension deficit^§^, in °	0.5 ± 1.5	0.5 ± 1.6	0.5 ± 1.6	0.9999
Dial test (IR) SSD^§^ in °	0 ± 5.7	− 0.5 ± 5.6	0.5 ± 6.0	0.7038
Dial test (AR) SSD^§^ in °	− 0.2 ± 3.9	− 0.7 ± 5.0	0.3 ± 2.5	0.1815
Rolimeter-Test^§^				
Lachman^§^ SSD, in mm	2 ± 1.6	2.7 ± 1.5	1.3 ± 1.3	**0.0339**
Stress Radiography (Telos®)				
ACL^§^ SSD in mm	1.8 ± 2.9	3.4 ± 2.2	0.4 ± 2.7	**0.0249**
PCL^§^ SSD in mm	4.3 ± 2.0	5.2 ± 2.2	3.6 ± 1.4	0.1048
Lachman test†				
Grade 1	10 (50)	5 (50)	5 (50)	n.s
Grade 2	4 (20)	4 (40)	0	0.0867
Grade 3	0	0	0	
Grade of pivot-shift test†				
Absent	19 (95)	9 (90)	10 (100)	n.s
Grade 1 (glide)	1 (5)	1 (10)	0	n.s
Grade 2 (clunk)	0	0	0	
Grade 3 (gross)	0	0	0	

Bold *p* value indicates statistical significance

*SD* standard deviation, *n.s.* not significant, *ACLIB* anterior cruciate ligament internal bracing, *ACLR* anterior cruciate ligament reconstruction

*n* = 20

^§^Mean ± SD

^†^*n* (in %)

^‡^Shapiro–Wilk normality test and Kolmogorov–Smirnov test were performed to determine if the data were normally distributed, to compare ACLIB and ACLR Students *t* test or Mann–Whitney *U* test were performed, Fisher’s exact test was used for comparison of binominal data

### Complications

Postoperative stiffness (Flexion < 90° and/or Extension deficit > 10°) was seen in seven cases (4 ACLIB and 3 ACLR), out of which were four type IV, two type IIIL and one type IIIM injuries. All patients did not respond to aggressive physiotherapy and therefore treated with early arthroscopic lysis of adhesions and debridement (LOA). After LOA all patients had a ROM greater 0/0/120° at final follow-up. In two cases disturbing endobuttons were removed simultaneously.

## Discussion

The main finding in this retrospective examination of acute knee dislocations was a superior anterior stability following ACL reconstruction compared to ACL repair, which was accompanied by a trend for improved patient-reported outcome scores. Clinical failure was observed in one patient of each group considering that a highly active patients were studied. A considerable rate of re-operation due to postoperative knee stiffness was noted in both groups with a higher remaining flexion deficit following ACL reconstruction. The treatment concept of the torn ACL in the setting of a multiligament knee injury has been a controversy since a long time [12, 25, 43]. Only a few studies reported about ACL reconstruction in acute knee dislocation using an early total repair strategy [10, 18, 32]. However, none of these studies performed a precise comparative analysis between ACL reconstruction and repair. It is known that in isolated ACL repair there is a considerable failure rate, especially when treating highly active patients with type III and IV ACL injuries according to Sherman classification [24, 36, 37]. In accordance, increased instrumented anterior–posterior laxity was reported in multiligamentary injuries compared to isolated ACL reconstruction [1–3, 11, 19, 23, 38]. Although correlation between PROMS and arthrometric results has been discussed controversially in isolated ACL reconstruction, an SSD of > 3 mm would be considered as an unsatisfactory result [14, 15, 40, 41]. Follow-up examinations in multiligament knee injuries frequently report SSD > 2 mm with a considerable trend towards higher values [8, 17–19]. In comparison to other studies, this study revealed lower laxity with ACL reconstruction, which was accompanied by a trend for improved clinical outcome scores without reaching significance. In line with our results, Hirschmann et al. reported a positive correlation of ACL reconstruction and clinical outcome [18]. Variance may be explained by additional bracing techniques and different types of ACL injury patterns as proximal tears seem to perform superior to midsubstance or distal tears [37, 42]. Single ACL repair and dynamic bracing resulted in 43% positive pivot-shift test and 2.5 mm SSD, but good Lysholm scores [23]. Internal bracing using a rigid suture augmentation resulted in 3.3 mm anterior–posterior SSD for the ACL and IKDC and Lysholm score > 80 [19], which was also reported by Rosteius et al. in close similarity [33]. These results are very similar to the outcomes using ACL repair in our study. Only few studies reported Lysholm scores > 85 as seen with ACL reconstruction in our study [12, 18, 19, 33]. In terms of graft failure 1/11 ACL graft rupture was observed in both groups, with the failure in both cases being due a traumatic event after return to sports. Both, graft failure and clinical outcome scores strongly depend on the patient age and activity level [22, 31, 34]. Average age of our study population was mid-age but with high active demands. Recent studies in isolated ACL reconstruction have shown higher graft failure rates in young and highly active patients, who perform high risk pivoting sports [34]. Therefore, the concept of ACL treatment in cases of acute KD may be chosen depending on the individual patient demand, with highly active and young patients having an advantage from ACL reconstruction. Included patients reported a preinjury Tegner level of 6, which can be considered as highly active. In line with previous reports, patients can return to a highly active sports level following acute knee dislocation [17]. 

From a biomechanical point of view less laxity of the cruciates may improve healing of the collaterals, which might explain the poorer results of staged surgery with peripheral fixation first and delayed cruciate reconstructions [21]. Rosteius reported about a considerable rate of residual laxity of the collaterals using the ACL repair strategy [33]. Animal models of combined ACL/MCL injuries using a robotic testing system have shown that initially high in situ forces within the ACL graft were transferred to the healing MCL during the early healing phase [26]. These excessive high loads likely contributed to a decrease in the structural properties of the MCL complex when compared to isolated MCL injuries [21].

Nevertheless, a considerable rate of stiffness and subsequent LOA in both groups has to be acknowledged. In comparison to a recently published systematic review [9], the rate of LOA was higher in our study as the indication for early LOA was made generous in this study. This decision was based on a recent study that revealed significantly improved range of motion and functional scores of early LOA (within 6 month) compared to late LOA (> 6 month) [7]. Although a postoperative flexion deficit of 10–15° has been reported before [27], a more progressive rehabilitation with unlimited range of motion may be necessary in future rehabilitation protocols.

Conclusions based on this study are limited by the relatively small case number and inhomogenous injury patterns. Improved comparability was tried to achieve by exclusion of obese patients, accompanying fractures of the tibial plateau, major nerve and vascular injuries. Decision for ACL treatment was not randomized, but changed during the study time with ACL reconstructions performed in the second half of the study period. In addition, no matched-pair analysis was feasible given the great rarity of acute KD. Long-term follow-up is necessary to validate the concept of primary ACL reconstruction as recent studies have shown the increase of graft failure during the observation time.

## Conclusion

Primary ACL reconstruction in type III/IV knee dislocations was shown to yield superior anterior objective knee stability in comparison to ACL repair and internal bracing in highly active patients and a trend for improved functional outcome scores was detected. Individualized ACL treatment within the concept of early complete repair may be necessary depending on the age and functional demands of the patient. Patients must be enlightened about the risk of flexion deficit and the need for LOA since both have a high prevalence in acute KD.

## Data Availability

The data that support the findings of this study are available on reasonable request from the corresponding author [MK]. The data are not publicly available due to containing information that could compromise the privacy of research participants.
